# Human-to-human transmission of leptospirosis: a global systematic review

**DOI:** 10.3389/fpubh.2026.1782163

**Published:** 2026-06-18

**Authors:** Kaya Stollberg, Martin Richter, Ulrika Windahl, Olena Pyskun, Johanna F. Lindahl, Martin Wainaina

**Affiliations:** 1 Department of Biological Safety, German Federal Institute for Risk Assessment, Berlin, Germany; 2 Department of Animal Health and Antimicrobial Strategies, Swedish Veterinary Agency, Uppsala, Sweden; 3 Department of Organization of Scientific and International Work, State Scientific and Research Institute of Laboratory Diagnostics and Veterinary and Sanitary Expertise, Kyiv, Ukraine; 4 Department of Medical Biochemistry and Microbiology, Zoonosis Science Center, Uppsala University, Uppsala, Sweden

**Keywords:** blood transfusion, breastfeeding, human-to-human transmission, *Leptospira*, person-to-person transmission, sexual transmission, systematic review, vertical transmission

## Abstract

**Introduction:**

Leptospirosis is typically transmitted when persons are exposed to infected animals or contaminated environments. Human-to-human transmission is currently reported non-systematically, and has unknown public health implications.

**Methods:**

Therefore, literature searches were performed on Embase, PubMed, Scopus, and Web of Science databases to identify relevant publications. Clinical data describing the index and secondary cases were extracted and the evidence strength was assessed.

**Results:**

The search yielded 10,658 results, and 27 publications covering 34 suspected reports were included. Strong evidence (*n* = 12) was found for vertical transmission occurring either transplacentally or via lactation. Transmission via sexual contact (*n* = 3) and blood transfusion (*n* = 1) was not well proven.

**Discussion:**

Human-to-human leptospirosis transmission may play a role in leptospiral epidemiology and lead to clinical cases, especially through vertical spread. However, no evidence of sustained human-to-human transmission was found as there was no record of infections beyond the secondary cases. Public health systems in endemic areas should remain vigilant for these less-reported modes of transmission to safeguard health. Robust One Health surveillance is required to improve detection and awareness, reduce underreporting and improve disease control.

## Introduction

Leptospirosis is a globally important zoonotic disease with over one million estimated human cases and 58,900 deaths annually (1). It is caused by bacteria of the genus *Leptospira* (2), which commonly infect animal hosts that can shed the pathogen in their urine. Small mammals, especially rats and other rodents, are important reservoirs for the pathogen. Infection in humans and animals occurs when bacteria gain entry through interrupted skin or mucous membranes. This can occur through direct or indirect contact with infected animals or contaminated soil or water either occupationally (e.g., slaughterhouse workers, veterinarians, paddy field farmers), or recreationally (e.g., swimming, kayaking) (3, 4). Humans are accidental hosts and infections can vary from asymptomatic to acute, with incubation periods between 3 and 30 days. Fulminant forms that involve multiple organs, e.g., leptospirosis-associated pulmonary haemorrhagic syndrome, meningitis/meningoencephalitis and Weil syndrome associated with jaundice, renal failure and myocarditis, can occur and often have high case fatality ratios (3, 5).

Leptospirosis is often underreported due to inadequate diagnostic capacity in endemic areas, and has a clinical presentation that initially often lacks distinguishing features from other endemic acute febrile illnesses such as brucellosis (6–8). Diagnosis is performed either directly using culture and PCR tests, or indirectly by detecting antibodies using serological tests such as the microscopic agglutination test (MAT) or ELISA (9).

There has been an increase in leptospirosis cases in various regions globally (10, 11). Extreme weather events such as flooding and heavy rainfall are thought to be important disease drivers, alongside population growth and urbanisation (12). Human-to-human transmission is a less-reported mode of transmission that has historically been given little attention in leptospiral epidemiology (13). However, with the dynamic nature of these disease drivers and risk factors for human infection, it remains unclear whether these rare modes of transmission could play more important roles in disease spread in the future. We therefore examined the literature to determine documented cases of transmission between persons and discuss the implications of the evidence to strengthen preparedness in the face of a changing disease landscape.

## Methods

The literature search was conducted on 24th September 2023 and updated on 31st March 2026 in leading databases which comprised Embase, PubMed, Scopus, and Web of Science. A review protocol was developed according to the Preferred Reporting Items for Systematic Reviews and Meta-Analyses (PRISMA) guidelines (14). The review was registered on OSF registries^1^ (15). The following search terms connected with Boolean operators were applied; (leptospira OR leptospirosis) AND (transfusion OR transplant OR vertical OR pregnan* OR human-to-human OR person-to-person OR sexual OR lactation OR aerosol OR mucosa) (Supplementary material). No limitations on language or publication time were placed in the searches.

The citations were imported into Endnote 21 (Clarivate Analytics, Philadelphia, PA, USA) and duplicates were removed. All citations were screened using the titles and abstracts for relevance, and the DeepL translation tool^2^ was used for non-English text. For initial screening, titles and abstracts were divided among all six authors, ensuring each record was independently reviewed by a second author. Discordant results were then resolved by MW. Two authors (KS and MW) conducted the data extraction from included publications.

Peer-reviewed journal articles that reported possible transmission between humans were included. All observational study designs (case reports, case series, cross-sectional, case–control, and longitudinal studies) were eligible, but secondary studies (literature reviews, systematic reviews, meta-analyses) were not included even though their reference sections were screened to obtain studies not found in the primary searches. All sampling methodologies (probabilistic and non-probabilistic) and census approaches were allowed. In cases of suspected vertical transmission but with no laboratory diagnosis, reports were excluded if it was clear that foetuses died from complications of maternal leptospirosis, or had positive outcomes with no indication of illness. Studies that reported leptospirosis in either index or secondary cases with the biological plausibility of transmission between persons were retained. Serological proof without accompanying clinical presentations consistent with leptospirosis was rejected on the basis of being too speculative to support potential transmission between people. The articles were screened independently by KS and MW and disagreements were discussed and resolved with co-authors.

Quality assessment was done according to a tailor-made tool designed to assess the strength of evidence for human-to-human transmission (Table 1). The use of a bespoke tool may have introduced subjectivity, but was aimed at gauging the strength of evidence for transmission between persons. We additionally assessed the quality of the included articles (study methodology) using the JBI Critical Appraisal tools for each study design^3^.

**Table 1:** A custom-made quality assessment tool used to gauge the strength of evidence for human-to-human transmission of leptospirosis.

Criteria	Criteria matched	Level of evidence	Interpretation
Domain 1 (D1): Laboratory-confirmed infection in both index and secondary cases*Domain 2 (D2): Clear epidemiological linkDomain 3 (D3): Temporal alignment between the two infectionsDomain 4 (D4): Transmission route is biologically plausible	4	Strong	High quality evidence of transmission between persons
	3	Moderate	Probable transmission but with incomplete evidence
	0–2	Weak	Anecdotal indication of transmission

^*^In cases of vertical transmission, laboratory-confirmed infections in foetuses or neonates (≤30 days) were enough to fulfil this criterion as environmental or zoonotic exposure was considered less likely.

Data analysis involved descriptive statistics and the creation of summary tables and figures.

## Results

### Database search results

**Figure 1:**
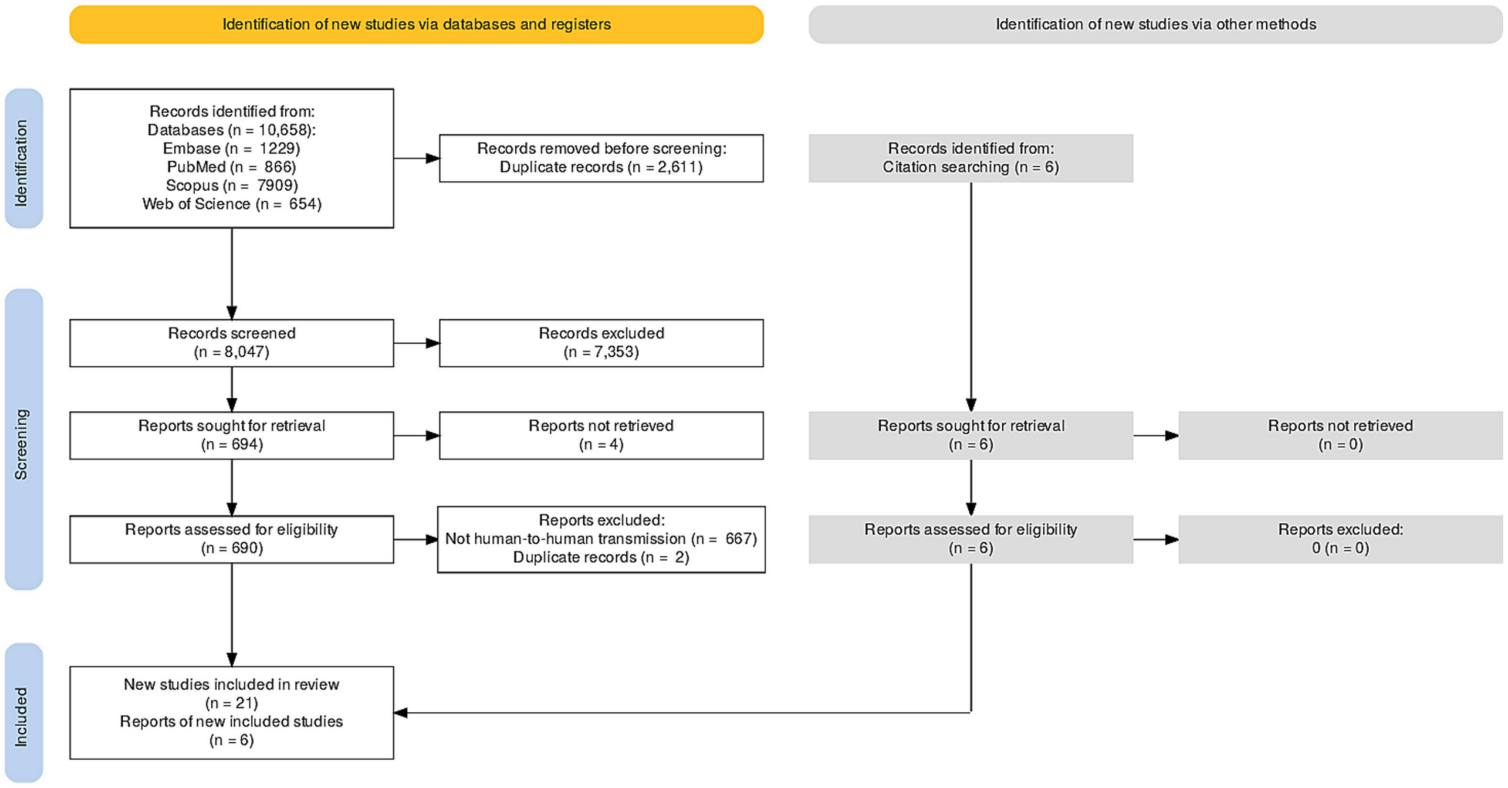
The study selection process for the systematic review utilised to identify relevant literature on human-to-human transmission of leptospirosis from leading electronic databases using PRISMA guidelines.

A total of 10,658 references were identified in the primary search, with Scopus giving 7,909 hits, and Embase (*n* = 1,229), PubMed (*n* = 866), and Web of Science (*n* = 654) following in decreasing order. The removal of duplicates from the combined search results gave 8,047 remaining references which were screened for relevance by title and abstract. Ultimately, 27 studies comprising 34 reports were considered relevant for this review (Figure 1).

### Study characteristics

The studies were published between 1932 and 2022, with most being in English (*n* = 17) and the rest in French (*n* = 3), German (*n* = 3), Chinese (*n* = 1), Dutch (*n* = 1), Hungarian (*n* = 1) and Spanish (*n* = 1). Most publications were from Europe (*n* = 10), followed by Asia (*n* = 8), South America (*n* = 4), Africa (*n* = 2), North America (*n* = 2) and Oceania (*n* = 1) in decreasing order. Lastly, most publications were case reports/series (*n* = 22), while cross-sectional (*n* = 4) and cohort (*n* = 1, originally labelled by authors as case–control) studies were fewer (Supplementary material).

### Quality assessment of evidence

**Figure 2:**
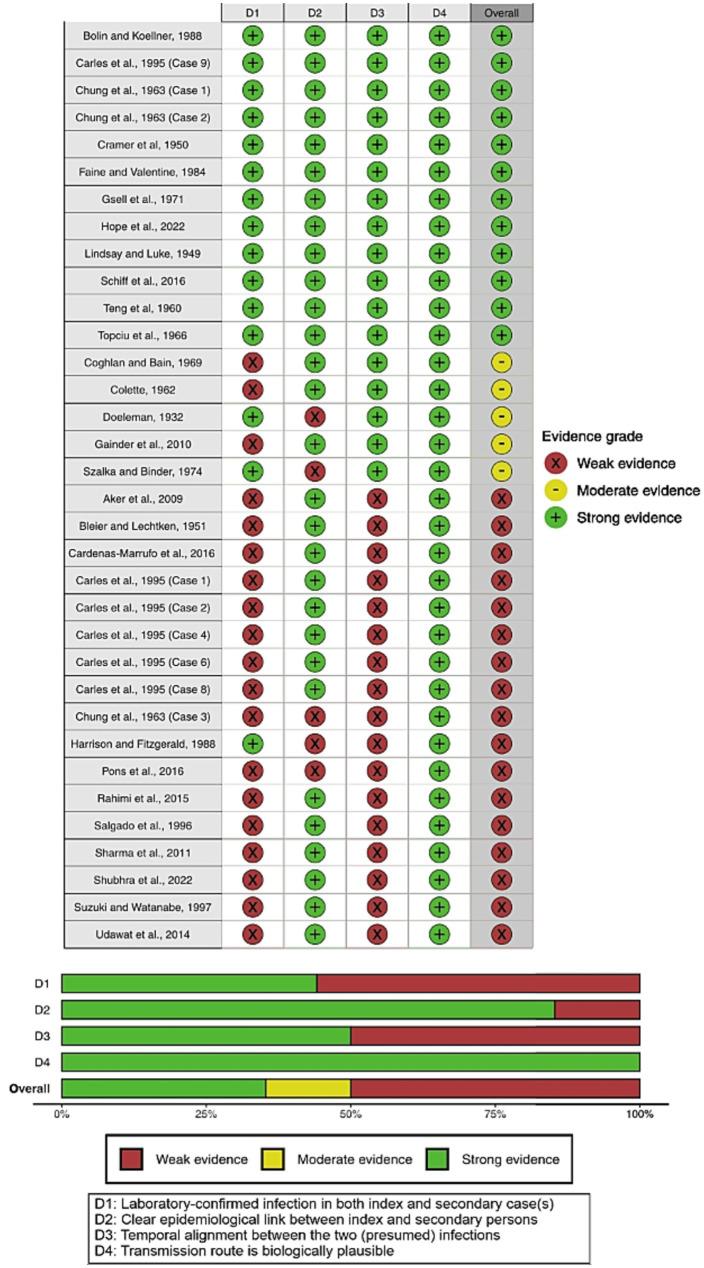
Assessment scores of the strength of evidence for transmission between persons based on four criteria from 34 reports. A summary bar chart is also presented with the results categorised into weak, moderate, and strong evidence of transmission.

The methodological assessment using the JBI critical appraisal tools showed that case reports met most criteria, with specific details on the interventions used being least reported. The case series and cohort study showed inadequate reporting of inclusion criteria, demographic details and statistical analyses. Prevalence studies diagnosed leptospirosis using well-established methods and described study settings sufficiently, but were weak on sampling methods, sample-size justification, and confidence interval reporting (Supplementary material). We further assessed the strength of the evidence for the transmission of leptospires between persons and revealed that overall, 12 reports (35.3%) had strong evidence of transmission, and 5 (14.7%) had moderate and 17 (50.0%) showed weak evidence (Figure 2).

### Vertical transmission

**Figure 3:**
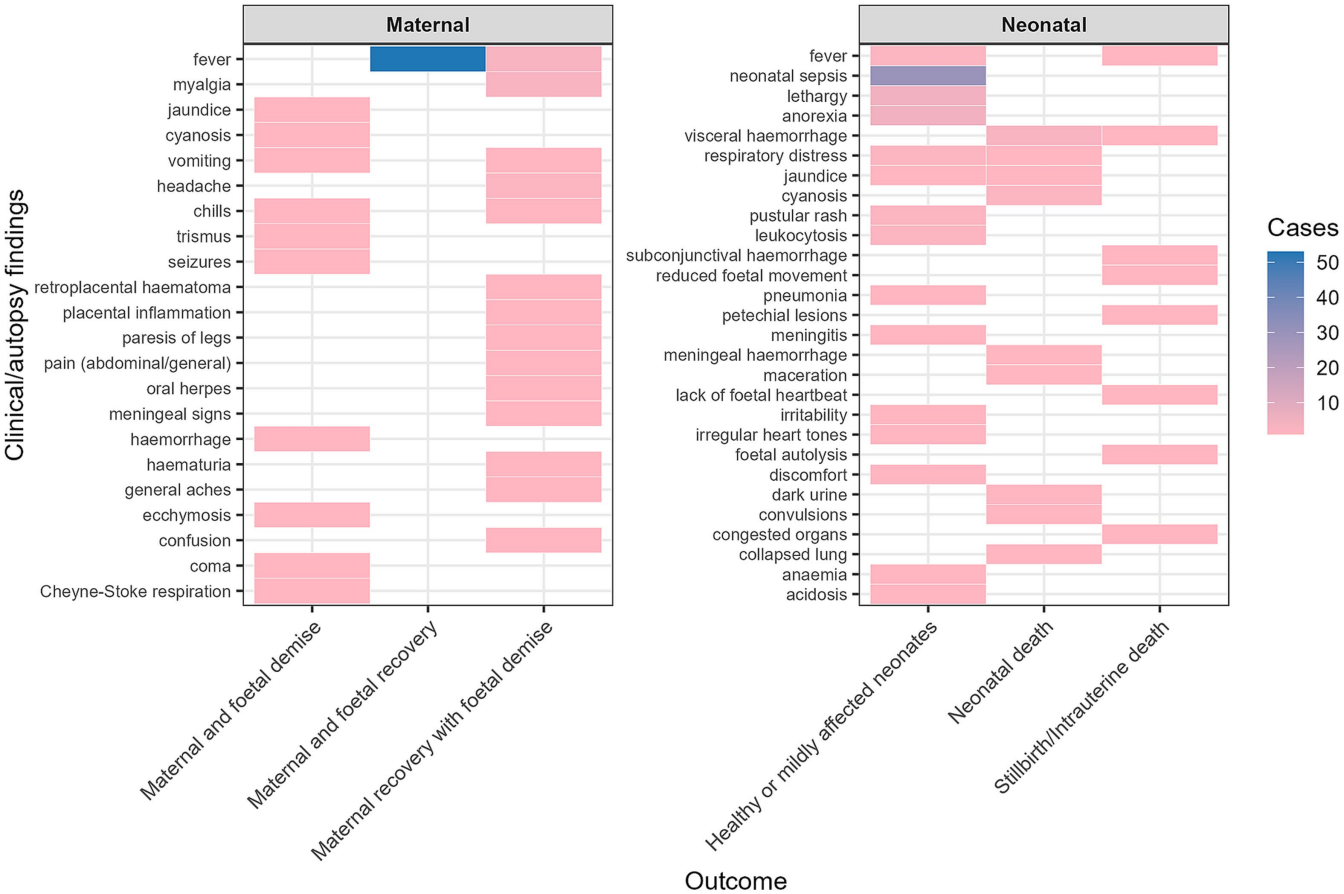
Heatmap of clinical/autopsy findings in infants and mothers with disease outcomes of cases of vertical leptospiral transmission with strong evidence as described in the respective studies.

Transmission from mother to child was indicated in 30 studies, among which 12 studies showed strong, 3 had moderate and 15 demonstrated weak evidence of transmission (Figure 2; Supplementary material). Most cases in the infant were acquired *in utero*, resulting in stillbirths/intrauterine death, and some cases were carried to term and resulted in neonatal death, or healthy/mildly affected neonates (Figure 3). Two cases were reportedly transmitted via breastfeeding: one was backed by strong evidence where leptospires were detected from both maternal and infant samples, and the other by weak evidence where leptospires were detected in breastmilk but without neonatal/infant testing. Sepsis was a commonly reported clinical manifestation in the affected neonates. Two studies lacked testing from maternal samples (Supplementary material), but *Leptospira* IgM antibodies and DNA were found in the neonates, indicating strong evidence of vertical transmission.

Additionally, maternal clinical findings were diverse and resulted in maternal recovery with foetal death or recovery (Figure 3). Fever, jaundice, myalgia and abdominal pain were common clinical presentations in infected mothers.

Laboratory confirmation was done using the MAT, ELISA, PCR, culture, or immunohistochemistry tests on maternal, infant, or paired maternal-neonate samples (Supplementary material). Where no laboratory testing was done on either index or secondary cases, clinical presentations or autopsy findings were relied upon to suspect transmission.

The reported risk factors associated with maternal exposure were occupational exposure to infected livestock through veterinary practice and farming, contact with infected pets, travel to endemic regions, farming in paddy fields, living near water/lowlands, exposure to rodent reservoirs, and swimming (Supplementary material).

### Sexual transmission

Three older case reports were found reporting sexual transmission of leptospirosis between sexual partners, and the evidence was of moderate to weak strength (Figure 2). Recreational exposure was a common risk factor in the index cases, and diagnosis was performed serologically using MAT and ELISA. Therefore, while sexual transmission was posited in the three reports, it was not proven by microbiological isolation of sexual fluids, bacterial typing beyond serovar identification, or robust contact tracing to ensure ruling out alternative sources of infection.

### Blood donation

A cross-sectional study was found demonstrating pathogenic/intermediate leptospires via PCR from 8/42 (19%) healthy blood donors in Cajamarca, Peru (Supplementary material). The area was described as rural with farming and exposure to rodent carriers as possible risk factors, and donors who were at risk of environmental and occupational exposure were included in the study, and thus not reflective of all blood donors. No transmission was established in blood recipients, but the demonstration of DNA in asymptomatic donors suggests subclinical carriage of the bacteria. The evidence for transmission was graded as weak (Supplementary material).

### Other transmission modes

No evidence was found for transmission between persons via aerosols and organ transplant.

## Discussion

We reviewed global literature spanning more than 90 years to investigate evidence of leptospirosis transmission between humans, a less reported route. Strong evidence was found for transmission from mother to child either via the placenta or breastfeeding. Moderate to weak evidence for sexual transmission was observed and weak evidence was found for transmission through blood transfusion. We discuss the implications of these biologically plausible routes for current public health policy and call for future studies with stronger evidence to better understand the risk of transmission and the impacts on disease prevention and control.

Entry of *Leptospira* during infection typically occurs through the interrupted skin and mucous membranes. Direct contact with the biological fluids of an infected animal such as urine, or indirect exposure to contaminated soil or water are responsible for most cases. Transmission between persons is biologically plausible via blood transfusion, tissue/organ transplant, vertically (during pregnancy or lactation), through sexual contact, through aerosols/droplets. However, sustained human-to-human transmission of zoonoses depends on the transmission route (e.g., airborne, contact, faecal-oral, and vector-borne transmission), host, pathogen, and environment (16, 17). Additionally, characteristics of the index and secondary persons (e.g., immune function, infectious dose), and transmission amplifiers that act between the index and secondary cases (e.g., climate, human behaviour, cultural variation) are important (17). Evidence from this review suggests that human-to-human transmission can lead to new clinical cases, but with little evidence of onward transmission beyond the secondary cases (sustained human-to-human transmission) which would have broader epidemiological consequences.

### Vertical transmission

Vertical transmission generally refers to the transfer of an aetiological agent from either parent to offspring, occurring mostly via the mother across the placenta (*in utero*), during childbirth (intrapartum), or after birth (postpartum) by breast-feeding (18). In our case, we observed mother-to-child transmission of leptospirosis occurring *in utero* (transplacentally) in most instances, with one case occurring via lactation with strong evidence. Vertical transmission has been demonstrated in some domestic animals, suggesting its likely importance in human hosts (19–23). Most publications were case reports or case series, which highlights the case-based nature of this transmission mode of leptospirosis, and need for stronger evidence through superior study designs with more statistical power (24). Stillbirths and intrauterine deaths, congenital abnormalities (e.g., hypotrophy), low birth weight, and small for gestational age were observed in neonates of leptospirosis-positive mothers. Fever, anaemia, jaundice and myalgia, as well as severe outcomes such as maternal mortality and pregnancy loss were common features of maternal leptospirosis, and were consistent with a recent comprehensive review of leptospirosis during pregnancy (25). Proving leptospirosis transmission during human gestation is not always conclusive, especially when there is no foetal/neonatal testing, a lack of obvious adverse foetal outcomes such as serious illness or death (26–34), or foetal death occurred from maternal complications from leptospirosis or otherwise (35–37). Therefore, it is likely our review introduced a selection bias by excluding unclear cases of vertical transmission which may underrepresent the real-world limitations of leptospirosis diagnostics. Additionally, histological proof of leptospires after foetal death was not always successful due to tissue maceration and likely death of leptospires, which can render test results inconclusive (38, 39). However, proper treatment and patient management are recommended as vertical transmission is likely underreported, especially in endemic areas, and a likely source of reporting bias in this review. Publication bias may also exist as unusual or severe outcomes are more likely to be published.

Lactation has been implicated as a possible transmission route in this review (40), a phenomenon observed in animal models too (41). The Chinese Medical Association has recommended the pasteurisation of breastmilk from infected mothers during treatment as it remains more beneficial to the infant than formula milk (42). Clinical suspicion of leptospirosis in endemic areas is critical, and boosting surveillance and diagnostics in perinatal care is important in reducing disease burden, especially of mild neonatal infections that are likely underreported in low-resource settings.

The diagnostic methods applied in the included studies investigating vertical transmission varied considerably. Older serological and histological methods can diagnose leptospirosis but not distinguish infective strains. For neonatal leptospirosis (≤7 days) however, the leptospirosis incubation period (3–30 days) (5), and the absence of environmental exposure *in utero* make infections from the mother the only biologically plausible source, irrespective of strain typing. For later infant cases however (8–30 days), the absence of molecular testing on mother and child samples means zoonotic and environmental sources of exposure cannot be fully ruled out.

### Sexual transmission

Diseases are sexually transmissible when the aetiological agent can be passed among persons via direct contact with interrupted skin, or through bodily fluids such as blood, semen and vaginal fluids during oral, anal, or genital sex with an infected partner (43). Additionally, categorising diseases as Sexually Transmitted Infections (STIs) requires sexual contact as the primary mode of transmission (43). Epidemiological comparisons of disease rates by sex, and molecular cluster analyses among sexually-linked samples can help recognise emerging STIs (44). The three studies positing sexual transmission of leptospirosis (45–47) were published between 1932 and 1988, when identifying leptospires largely relied on serology and culture methods, and no PCR assays were available yet (48). Consequently, no leptospires were demonstrated in the patients’ bodily fluids associated with sexual transmission, and only circumstantial evidence was provided, e.g., acute disease after sexual contact with partner with known exposure to partner with no discernible risk factor. This moderate evidence makes us conclude that sexual transmission of leptospirosis is currently not well proven. Studies are required to demonstrate leptospires and their survival in semen, vaginal fluids, genital and rectal swabs, etc. from infected persons, and molecular analyses showing the phylogenetic relationship between linked cases are required to improve the understanding of this transmission mode. Providing detailed exposure history through contact tracing could help track cases, but using this approach is limited in STIs due to its associated intrusive nature and stigmatisation (49). Ruling out other sources of exposure by animal and environmental sampling is critical in strengthening evidence of sexual transmission of leptospirosis. However, venereal leptospiral transmission has been observed in various domestic animals and murine models (21, 50–53), and experimental infection of laboratory models through the vaginal and anal routes has been demonstrated, therefore showing the biological plausibility in humans (54). Experimental oral infection has also been demonstrated, but the route may be inefficient due to protection from saliva, intact oral mucosa and gastric acid (54, 55).

### Transfusion-mediated transmission

Various emerging and re-emerging pathogens that are not part of standard screening pose a risk to transfusion medicine (56). The included study demonstrated leptospiral DNA in donated blood from high-risk donors in an endemic area in Peru, offering credible evidence of potential transmission (57). Several studies demonstrating past infections in blood donors using serology also exist, showing the potential risk of transmission via blood transfusion (58–62). Bacteraemia is often associated with the initial leptospiraemic phase during the first 8 days of fever (3), but PCR positivity in asymptomatic blood donors suggests subclinical bacterial carriage, the risk of transmission of which is not clear. Eligibility criteria for blood donation that filter out febrile syndrome would help curb transmission via transfusion of blood and blood products. However, blood donor eligibility varies substantially across nations, and criteria that are evidence-based are recommended (63). Pathogen reduction technologies for donated blood such as irradiation could also play a role in reducing transmission of emerging pathogens (56). Artificial oxygen carriers (artificial blood) are novel approaches that may help prevent transmission of emerging transfusion-mediated infections, especially in those requiring repeat transfusions such as sickle cell anaemia and thalassemia patients. However, these therapies are not yet approved for use in several countries due to high toxicities (64, 65).

### Organ transplant-mediated transmission

No reports of transmission via organ transplants were found. However, solid organ transplant guidelines in the endemic south Asian region have recommended leptospirosis screening in organ donors and recipients with significant epidemiological risk or exposure due to the possibility of transplant-mediated leptospirosis (66). Additionally, donation should be deferred to 3 months after recovery, and treatment in recipients should follow protocols used for immunosuppressed patients (66). Case reports of liver and kidney transplant recipients who acquired leptospirosis due to immunosuppressed states have been reported, and the infections were attributed to contaminated environments and not donor-derived (67, 68).

### Aerosol transmission

No reports of airborne transmission were found. Pulmonary involvement in leptospirosis is well documented, and characterised by chest pain, cough, dyspnoea and haemoptysis. Acute respiratory distress syndrome (ARDS) and severe pulmonary haemorrhagic syndrome (SPHS) are severe forms of leptospirosis that can lead to respiratory insufficiency and death (69, 70). Leptospires have also been detected in respiratory samples of patients with sudden worsening of chronic obstructive pulmonary disease (COPD) by amplicon sequencing of endotracheal aspirates (71). Hamster models have shown that leptospires have a predilection for lungs as evidenced by pulmonary lesions, haemorrhage, and elevated inflammation markers after experimental inoculation (72). Consequently, the possibility of transmission via droplets from ill patients to surrounding persons cannot be ruled out. However, few leptospires are found in lung tissue despite severe pulmonary injury, and pulmonary manifestations are likely from bacterial toxins or host immune response and not from local bacterial replication (69, 70). Laboratory models (guinea pigs and rats) that were infected intranasally all survived and had no signs of infection, which suggests that this route may be inefficient (54).

## Conclusion

In conclusion, we provide a comprehensive synthesis of reported cases of human-to-human transmission of leptospirosis. Exposure to contaminated environments and infected animals remain the most important routes of acquiring leptospirosis in humans. In addition, credible evidence of vertical transmission, both transplacentally and via breastfeeding, was found. Evidence for sexual transmission was moderate to weak. Possible risk of transmission through blood transfusion was found (weak evidence), and no reports of transmission via solid organ transplantation or aerosols between persons were found, despite these routes being biologically plausible. These findings demonstrate the need to consider leptospirosis in maternal and neonatal healthcare, especially in endemic and outbreak-prone areas. As risk factors for leptospiral exposure in the index cases of infections found in this review were zoonotic or environmental, robust surveillance systems that utilise integrated One Health approaches are required (73). There may be need for targeted testing of at-risk populations (such as pregnant mothers and blood donors) in endemic countries (74), but routine population-level human-to-human transmission surveillance may not be justified based on this evidence. Applying molecular diagnostics to determine the traceability of cases will help elucidate human-to-human transmission of leptospirosis and better understand the risks posed to human health, especially in transplant and transfusion medicine, sexual health, and maternal and neonatal health.

## Data Availability

The original contributions presented in the study are included in the article/Supplementary material, further inquiries can be directed to the corresponding author.

## Footnotes

1
https://osf.io/3rjck

2
https://www.deepl.com/en/translator

3
https://jbi.global/critical-appraisal-tools

## References

[1] Costa F Hagan JE Calcagno J Kane M Torgerson P Martinez-Silveira MS . Global morbidity and mortality of leptospirosis: a systematic review. PLoS Negl Trop Dis. (2015) 9:e0003898. doi: 10.1371/journal.pntd.0003898, 26379143 PMC4574773

[2] Levett PN. "*Leptospira*". In: Jorgensen JH Carroll KC Funke G Pfaller MA Landry ML Richter SS , editors. Manual of Clinical Microbiology. Washington DC: ASM Press (2015). p. 1028–36.

[3] Haake DA Levett PN. "Leptospirosis in humans". In: Adler B, editor. *Leptospira* and Leptospirosis. Springer Berlin Heidelberg: Berlin, Heidelberg (2015). p. 65–97. 10.1007/978-3-662-45059-8_5 PMC4442676 25388133

[4] Baharom M Ahmad N Hod R Ja’afar MH Arsad FS Tangang F . Environmental and occupational factors associated with leptospirosis: a systematic review. Heliyon. (2024) 10:e23473. doi: 10.1016/j.heliyon.2023.e23473, 38173528 PMC10761560

[5] Sykes JE Haake DA Gamage CD Mills WZ Nally JE. A global one health perspective on leptospirosis in humans and animals. J Am Vet Med Assoc. (2022) 260:1589–96. doi: 10.2460/javma.22.06.0258, 35895801

[6] Wainaina M Vey da Silva DA Dohoo I Mayer-Scholl A Roesel K Hofreuter D . A systematic review and meta-analysis of the aetiological agents of non-malarial febrile illnesses in Africa. PLoS Negl Trop Dis. (2022) 16:e0010144. doi: 10.1371/journal.pntd.0010144, 35073309 PMC8812962

[7] Wainaina M Wasonga J Cook EAJ. Epidemiology of human and animal leptospirosis in Kenya: a systematic review and meta-analysis of disease occurrence, serogroup diversity and risk factors. PLoS Negl Trop Dis. (2024) 18:e0012527. doi: 10.1371/journal.pntd.0012527, 39331677 PMC11463743

[8] Wainaina M Kimatu JS Rukwaro B Cook EAJ. Epidemiology of human and animal brucellosis in Kenya: a One Health meta-regression and network analysis. One Health. (2026) 22:101390. doi: 10.1016/j.onehlt.2026.101390, 41938085 PMC13049966

[9] WOAH. "Leptospirosis". In: World Organisation for Animal Health, editor. Manual of Diagnostic Tests and Vaccines for Terrestrial Animals. 13th ed.: World Organisation for Animal Health (2021)

[10] Muñoz-Zanzi C Groene E Morawski BM Bonner K Costa F Bertherat E . A systematic literature review of leptospirosis outbreaks worldwide, 1970–2012. Rev Panam Salud Publica. (2020) 44:e78. doi: 10.26633/RPSP.2020.78, 32684917 PMC7363284

[11] Beauté J Innocenti F Aristodimou A Špačková M Eves C Kerbo N . Epidemiology of reported cases of leptospirosis in the EU/EEA, 2010 to 2021. Eurosurveillance. (2024) 29:2300266. doi: 10.2807/1560-7917.ES.2024.29.7.2300266, 38362624 PMC10986659

[12] Lau CL Smythe LD Craig SB Weinstein P. Climate change, flooding, urbanisation and leptospirosis: fuelling the fire? Trans R Soc Trop Med Hyg. (2010) 104:631–8. doi: 10.1016/j.trstmh.2010.07.002, 20813388

[13] Rajapakse S Fernando N Dreyfus A Smith C Rodrigo C. Leptospirosis. Nat Rev Dis Prim. (2025) 11:32. doi: 10.1038/s41572-025-00614-5, 40316520

[14] Page MJ McKenzie JE Bossuyt PM Boutron I Hoffmann TC Mulrow CD . The PRISMA 2020 statement: an updated guideline for reporting systematic reviews. BMJ. (2021) 372:n71. doi: 10.1136/bmj.n71, 33782057 PMC8005924

[15] Wainaina M. Human-to-human Transmission of Leptospirosis: A Scoping Review. OSF Registries (2024). doi: 10.17605/OSF.IO/3RJCK

[16] Richard M Knauf S Lawrence P Mather AE Munster VJ Müller MA . Factors determining human-to-human transmissibility of zoonotic pathogens via contact. Curr Opin Virol. (2017) 22:7–12. doi: 10.1016/j.coviro.2016.11.004, 27907884 PMC5346033

[17] de Graaf M Beck R Caccio SM Duim B Fraaij PLA Le Guyader FS . Sustained fecal-oral human-to-human transmission following a zoonotic event. Curr Opin Virol. (2017) 22:1–6. doi: 10.1016/j.coviro.2016.11.001, 27888698 PMC7102779

[18] Cason J Mant CA. High-risk mucosal human papillomavirus infections during infancy & childhood. J Clin Virol. (2005) 32:52–8. doi: 10.1016/j.jcv.2004.12.007, 15753012

[19] Nogueira DB da Costa FTR de Sousa Bezerra C Soares RR da Costa Barnabé NN Falcão BMR . *Leptospira* sp. vertical transmission in ewes maintained in semiarid conditions. Anim Reprod Sci. (2020) 219:106530. doi: 10.1016/j.anireprosci.2020.106530, 32828405

[20] Magajevski FS Gírio RJS Meirelles RB. Pesquisa de *Leptospira* em fetos de vacas abatidas no estado São Paulo, Brasil. Arquivos do Instituto Biológico. (2007) 74:67–72.

[21] Cilia G Bertelloni F Piredda I Ponti MN Turchi B Cantile C . Presence of pathogenic *Leptospira* spp. in the reproductive system and fetuses of wild boars (*Sus scrofa*) in Italy. PLoS Negl Trop Dis. (2020) 14:e0008982. doi: 10.1371/journal.pntd.0008982, 33370309 PMC7793250

[22] Terayama Y Maeda M Hata Y Kawasaki Y Koizumi N. A probable cluster of premature birth and stillbirth caused by *Leptospira interrogans* serogroup Hebdomadis in an integrated swine farm in Nagasaki prefecture, Japan. J Vet Med Sci. (2024) 86:1040–4. doi: 10.1292/jvms.24-0215, 39111848 PMC11442401

[23] da Costa Barnabé NN Soares RR Barros DK Araújo Júnior JP Malossi CD Rodrigues Silva ML . The role of transplacental infection in *Leptospira* spp. epidemiology in cattle in Caatinga biome, Brazil. Microorganisms. (2024) 12. doi: 10.3390/microorganisms12061044, 38930426 PMC11205532

[24] Murad MH Asi N Alsawas M Alahdab F. New evidence pyramid. Evidence Based Med. (2016) 21:125–7. doi: 10.1136/ebmed-2016-110401, 27339128 PMC4975798

[25] Selvarajah S Ran S Roberts NW Nair M. Leptospirosis in pregnancy: a systematic review. PLoS Negl Trop Dis. (2021) 15:e0009747. doi: 10.1371/journal.pntd.0009747, 34520461 PMC8462732

[26] Chedraui PA San Miguel G. A case of leptospirosis and pregnancy. Arch Gynecol Obstet. (2003) 269:53–4. doi: 10.1007/s00404-002-0415-3, 14605821

[27] Dadhwal V Bahadur A Deka D. Leptospirosis as a cause of fever in pregnancy. Int J Gynecol Obstet. (2007) 99:252–3. doi: 10.1016/j.ijgo.2007.05.036, 17888438

[28] Gaspari R Annetta MG Cavaliere F Pallavicini F Grillo R Conti G . Unusual presentation of leptospirosis in the late stage of pregnancy. Minerva Anestesiol. (2007) 73:429–32. 17637589

[29] Hicham S Ihsane M El Bouazzaoui A Brahim B Labib S Mustapha H . Multivisceral organ failure related to leptospirosis in pregnant patient. Indian J Crit Care Med. (2013) 17:43–5. doi: 10.4103/0972-5229.112143, 23833476 PMC3701397

[30] Koe SL Tan KT Tan TC. Leptospirosis in pregnancy with pathological fetal cardiotocography changes. Singapore Med J. (2014) 55:e20–4. doi: 10.11622/smedj.2013194, 24712035 PMC4291937

[31] Patel T Pajai S Patel N Patel P. Leptospirosis manifesting as HELLP (hemolysis, elevated liver enzymes, and Low platelets) syndrome: a rare case of leptospirosis during pregnancy. Cureus. (2023) 15:e39083. doi: 10.7759/cureus.39083, 37332452 PMC10269396

[32] Rathnaweera R. A death of a pregnant mother following leptospirosis. Medico-Legal J Sri Lanka. (2015) 1:20–2. doi: 10.4038/mljsl.v1i3.7303

[33] Shaked Y Shpilberg O Samra D Samra Y. Leptospirosis in pregnancy and its effect on the fetus: case report and review. Clin Infect Dis. (1993) 17:241–3. doi: 10.1093/clinids/17.2.241, 8399874

[34] Tayade S Madaan S Kumar S Talwar D Chadha A. Tropical infections induced fulminant hepatitis in peripartum managed successfully: Tales of fate. Cureus. (2022) 14:e22223. doi: 10.7759/cureus.22223, 35340480 PMC8928236

[35] Baytur YB Cabuk M Koyuncu FM Lacin S Ceylan C Kandiloglu AR. Weil's syndrome in pregnancy. Eur J Obstet Gynecol Reprod Biol. (2005) 119:132–3. doi: 10.1016/j.ejogrb.2004.06.021, 15734102

[36] Sutter T Fehr T Blume C Kajdi M-E. Stillbirth and fulminant postpartum haemolysis: COVID-19 or leptospirosis or both? BMJ Case Rep. (2023) 16:e252620. doi: 10.1136/bcr-2022-252620, 37399345 PMC10314429

[37] Tramoni G Clément HJ Lopez F Viale JP. An unusual case of post partum haemorrhage: leptospirosis infection. Annales Françaises d'Anesthésie et de Réanimation. (2003) 22:363–5. doi: 10.1016/S0750-7658(03)00061-3, 12818331

[38] Coghlan JD Bain AD. Leptospirosis in human pregnancy followed by death of the foetus. Br Med J. (1969) 1:228–30. doi: 10.1136/bmj.1.5638.228, 5762626 PMC1982050

[39] Colette C. Fatal leptospirosis during pregnancy with severe jaundice. Bull Fed Soc Gynecol Obstet Lang Fr. (1962) 14:437–40. 14022281

[40] Bolin CA Koellner P. Human-to-human transmission of *Leptospira interrogans* by milk. J Infect Dis. (1988) 158:246–7. doi: 10.1093/infdis/158.1.246, 3392418

[41] De Oliveira D Figueira CP Zhan L Pertile AC Pedra GG Gusmão IM . *Leptospira* in breast tissue and milk of urban Norway rats (*Rattus norvegicus*). Epidemiol Infect. (2016) 144:2420–9. doi: 10.1017/S0950268816000637, 27019024 PMC5437553

[42] Yihua Z Huixia Y Xinghui L. Expert consensus on breastfeeding in case of maternal infections. Soc. Perinatal Med Chinese Med Association. (2021) 24:481–489. doi: 10.3760/cma.j.cn113903-20210530-00507

[43] Allan-Blitz L-T Gandhi M Adamson P Park I Bolan G Klausner JD. A position statement on Mpox as a sexually transmitted disease. Clin Infect Dis. (2023) 76:1508–12. doi: 10.1093/cid/ciac960, 36546646 PMC10110265

[44] Bernstein K Bowen VB Kim CR Counotte MJ Kirkcaldy RD Kara E . Re-emerging and newly recognized sexually transmitted infections: can prior experiences shed light on future identification and control? PLoS Med. (2017) 14:e1002474. doi: 10.1371/journal.pmed.1002474, 29281630 PMC5744912

[45] Harrison NA Fitzgerald WR. Leptospirosis — can it be a sexually transmitted disease? Postgrad Med J. (1988) 64:163–4. doi: 10.1136/pgmj.64.748.163, 3174532 PMC2428802

[46] Szalka A Binder L. A rare case of human-to-human infection of leptospirosis. Orv Hetil. (1974) 115:1531–2. 4840706

[47] Doeleman F. Ziekte van Weil, rechstreeks overgebracht van mensch op mensch. Ned Tijdschr Geneeskd. (1932) 76:5057–8.

[48] Van Eys GJ Gravekamp C Gerritsen MJ Quint W Cornelissen MT Schegget JT . Detection of leptospires in urine by polymerase chain reaction. J Clin Microbiol. (1989) 27:2258–62. doi: 10.1128/jcm.27.10.2258-2262.1989, 2584377 PMC267006

[49] Brandt AM. The history of contact tracing and the future of public health. Am J Public Health. (2022) 112:1097–9. doi: 10.2105/AJPH.2022.306949, 35830671 PMC9342804

[50] Soares RR da Costa Barnabé NN Júnior JPA Malossi CD Ullmann LS da Costa DF . Investigation of the presence of *Leptospira interrogans* in urinary and genital tracts of male goats raised in the semiarid region of Brazil. Small Rumin Res. (2023) 218:106880. doi: 10.1016/j.smallrumres.2022.106880

[51] Hamond C Martins G Medeiros MA Lilenbaum W. Presence of Leptospiral DNA in semen suggests venereal transmission in horses. J Equine Vet Sci. (2013) 33:1157–9. doi: 10.1016/j.jevs.2013.03.185

[52] Loureiro AP Lilenbaum W. Genital bovine leptospirosis: a new look for an old disease. Theriogenology. (2020) 141:41–7. doi: 10.1016/j.theriogenology.2019.09.011, 31518727

[53] Shetty A Kundu S Vernel-Pauillac F Ratet G Werts C Gomes-Solecki M. Transient presence of live *Leptospira interrogans* in murine testes. Microbiol Spectr. (2022) 10:e02775–21. doi: 10.1128/spectrum.02775-21, 35446113 PMC9241917

[54] Stavitsky AB. Studies on the mechanism of host resistance in experimental leptospirosis Icterohemorrhagica. J Immunol. (1945) 51:397–419. doi: 10.4049/jimmunol.51.6.397, 21011040

[55] Asoh T Saito M Villanueva SYAM Kanemaru T Gloriani N Yoshida S-i. Natural defense by saliva and mucosa against oral infection by *Leptospira*. Can J Microbiol. (2014) 60:383–9. doi: 10.1139/cjm-2014-0016, 24861456

[56] Cardoso M Ragan I Hartson L Goodrich RP. Emerging pathogen threats in transfusion medicine: improving safety and confidence with pathogen reduction technologies. Pathogens. (2023) 12. doi: 10.3390/pathogens12070911, 37513758 PMC10383627

[57] Pons MJ Urteaga N Alva-Urcia C Lovato P Silva J Ruiz J . Infectious agents, *Leptospira* spp. and *Bartonella* spp., in blood donors from Cajamarca, Peru. Blood Transfus. (2016) 14:504–8. doi: 10.2450/2015.0081-15, 26674831 PMC5111371

[58] Faddy H Seed C Lau C Racloz V Flower R Smythe L . Antibodies to *Leptospira* among blood donors in higher-risk areas of Australia: possible implications for transfusion safety. Blood Transfus. (2015) 13:32–6. doi: 10.2450/2014.0012-14, 24960651 PMC4317087

[59] Ribeiro MA Cliquet MG Santos MGS. Leptospirosis: a problem for transfusion medicine? Serodiagn Immunother Infect Dis. (1997) 8:185–9. doi: 10.1016/S0888-0786(96)01076-1, 36375451

[60] Limothai U Tachaboon S Dinhuzen J Singh J Jirawannaporn S Leewongworasingh A . Seroprevalence of leptospirosis among blood donors in an endemic area. Sci Rep. (2023) 13:12336. doi: 10.1038/s41598-023-39461-3, 37524788 PMC10390486

[61] Nedunchelliyan S Venugopalan AT. Blood transfusion and leptospirosis. Indian Vet J. (1997) 74:790–1.

[62] Chenna D Shastry S Singson S Balasubramanian R Bhatia A. Unveiling *Leptospira* antibody seroprevalence among voluntary blood donors: insights from a single-center observational study. J Appl Hematol. (2024) 15:228–32. doi: 10.4103/joah.joah_38_24, 42163182

[63] Karp JK King KE. International variation in volunteer whole blood donor eligibility criteria. Transfusion. (2010) 50:507–13. doi: 10.1111/j.1537-2995.2009.02392.x, 19788512

[64] Khan F Singh K Friedman MT. Artificial blood: The history and current perspectives of blood substitutes. Discoveries (Craiova). (2020) 8:e104. doi: 10.15190/d.2020.1, 32309621 PMC7086064

[65] Mohanto N Park Y-J Jee J-P. Current perspectives of artificial oxygen carriers as red blood cell substitutes: a review of old to cutting-edge technologies using in vitro and in vivo assessments. J Pharm Investig. (2023) 53:153–90. doi: 10.1007/s40005-022-00590-y, 35935469 PMC9344254

[66] Bansal SB Ramasubramanian V Prasad N Saraf N Soman R Makharia G . South Asian transplant infectious disease guidelines for solid organ transplant candidates, recipients, and donors. Transplantation. (2023) 107:1910–34. doi: 10.1097/TP.0000000000004521, 36749281

[67] Song ATW Abas L Andrade LC Andraus W D'Albuquerque LAC Abdala E. A first report of leptospirosis after liver transplantation. Transpl Infect Dis. (2016) 18:137–40. doi: 10.1111/tid.12490, 26671230

[68] Gerasymchuk L Swami A Carpenter CF Samarapungavan D Batke M Kanhere R . Case of fulminant leptospirosis in a renal transplant patient. Transpl Infect Dis. (2009) 11:454–7. doi: 10.1111/j.1399-3062.2009.00415.x, 19558375

[69] Dolhnikoff M Mauad T Bethlem EP Carvalho CRR. Pathology and pathophysiology of pulmonary manifestations in leptospirosis. Braz J Infect Dis. (2007) 11:142–8. doi: 10.1590/S1413-86702007000100029, 17625743

[70] Gulati S Gulati A. Pulmonary manifestations of leptospirosis. Lung India. (2012) 29:347–53. doi: 10.4103/0970-2113.102822, 23243349 PMC3519021

[71] Huang YJ Kim E Cox MJ Brodie EL Brown R Wiener-Kronish JP . A persistent and diverse airway microbiota present during chronic obstructive pulmonary disease exacerbations. OMICS: A Journal of Integrative Biology. (2010) 14:9–59. doi: 10.1089/omi.2009.0100, 20141328 PMC3116451

[72] Marinho M Oliveira-Júnior IS Monteiro CMR Perri SH Salomão R. Pulmonary disease in hamsters infected with *Leptospira interrogans*: histopathologic findings and cytokine mRNA expressions. Am J Trop Med Hyg. (2009) 80:832–6. doi: 10.4269/ajtmh.2009.80.832 19407133

[73] Muñoz-Zanzi C Dreyfus A Limothai U Foley W Srisawat N Picardeau M . Leptospirosis—improving healthcare outcomes for a neglected tropical disease. Open Forum Infect Dis. (2025) 12:ofaf035. doi: 10.1093/ofid/ofaf035, 39963696 PMC11832045

[74] Sandoval KL Cada KJS Dimasin RVD Labana RV. A one health approach to the prevention, control, and management of leptospirosis: a scoping review. Discover Public Health. (2025) 22:108. doi: 10.1186/s12982-025-00489-7

