# Supplementary material for: Human-to-human transmission of leptospirosis: a global systematic review
Source: Front Public Health. 2026 Jun 18;14:1782163. doi: 10.3389/fpubh.2026.1782163 (PMC13323498; doi:10.3389/fpubh.2026.1782163)
Supplement: Supplementary file 1 [file Supplementary_file_1.docx]

**Human-to-human transmission of leptospirosis: A global systematic review**

Kaya Stollberg, Martin Richter, Ulrika Windahl, Olena Pyskun, Johanna F. Lindahl, Martin Wainaina

**Contents**

[**1.** **Search terms** 1](#_Toc230165759)

[**2.** **Study characteristics** 3](#_Toc230165760)

[**3.** **JBI Quality assessment** 12](#_Toc230165761)

[**4.** **Supplementary references** 15](#_Toc230165762)

[**5.** **PRISMA Checklist** 17](#_Toc230165763)

Literature searches were conducted on 24.09.2023 and updated on 31.03.2026.

# **Search terms**

Supplementary Table 1: A summary of the search terms used for literature identification

| **Search terms (All fields where setting is available)** | **Database** | **Results** | | |
| --- | --- | --- | --- | --- |
|  |  | **Original** | **Update** | **Total** |
| (leptospira OR leptospirosis) AND (transfusion OR transplant OR vertical OR pregnan* OR human-to-human OR person-to-person OR sexual OR lactation OR aerosol OR mucosa) | PubMed | 779 | 87 | 866 |
| (leptospira OR leptospirosis) AND (transfusion OR transplant OR vertical OR pregnan* OR human-to-human OR person-to-person OR sexual OR lactation OR aerosol OR mucosa) | Scopus | 6085 | 1824 | 7909 |
| ('leptospira'/exp OR leptospira OR 'leptospirosis'/exp OR leptospirosis) AND (transfusion OR transplant OR vertical OR pregnan* OR 'human to human' OR 'person to person' OR sexual OR lactation OR aerosol OR mucosa) | Embase | 996 | 233 | 1229 |
| (leptospira OR leptospirosis) AND (transfusion OR transplant OR vertical OR pregnan* OR human-to-human OR person-to-person OR sexual OR lactation OR aerosol OR mucosa) (Topic) | Web of Science  (All collections, i.e.,  Web of Science Core Collection, Biological Abstracts, BIOSIS Citation Index, BIOSIS Previews, CABI: CAB Abstracts®, Current Contents Connect, Data Citation Index, Derwent Innovations Index, Grants Index, KCI-Korean Journal Database, MEDLINE®, Policy Citation Index, ProQuest ™ Dissertations & Theses Citation Index, SciELO Citation Index, Zoological Record) | 397 | 257 | 654 |
| **Total** |  | **8257** | **2401** | **10658** |

# **Study characteristics**

Supplementary Table 2: A summary of study characteristics of publications investigating human-to-human transmission of leptospirosis globally. Studies are presented according to the mode of transmission and evidence grade determined.

|  |  |  | **Index case(s)** | | | **Secondary case(s)** | | |  |  |
| --- | --- | --- | --- | --- | --- | --- | --- | --- | --- | --- |
| **Article** | **Country** | **Mode of transmission** | **Diagnostic test** | **Samples** | **Results (species/serovar found) *** | **Diagnostic test** | **Samples** | **Results *** | **Interventions and outcomes** | **Evidence Grade** |
| Carles et al., 1995  (Case 9) [1] | French Guiana | Vertical (transplacental) | Culture, serology | Blood, urine | *L. interrogans* serovar Icterohaemorrhagiae | Culture | Placenta | *Leptospira* spp. | Mother treated using ampicillin. Pregnancy resulted in intrauterine foetal death. | Strong |
| Chung et al., 1963  (Case 1) [2] | China | Vertical (transplacental) | Culture, animal inoculation | Blood | *L. borgpetersenii* serovar Hardjo (Hardjobovis) | Culture, animal inoculation, CFT | Placenta, blood, amniotic fluid | *Leptospira* spp. | Neonate treated with penicillin (with vitamins K and C supplements) and recovered. No maternal interventions specified but she recovered. | Strong |
| Chung et al., 1963  (Case 2) [2] | China | Vertical (transplacental) | Culture | Blood | *Leptospira* spp. | Culture | Liver, kidney | *Leptospira* spp. | Mother was hospitalised for leptospirosis treatment. Abortion occurred on night of admission. | Strong |
| Cramer et al, 1950 [3] | Germany | Vertical (transplacental) | Agglutination test | Blood, CSF, urine | *L. interrogans* serovar Canicola | Levaditi stain | Placenta | *Leptospira* spp. | Symptomatic management on mother after illness resulting in spontaneous abortion. She recovered and was discharged. | Strong |
| Faine and Valentine, 1984 [4] | Australia | Vertical (transplacental) | Serology | Serum | *L. interrogans* serovar Hardjo | Immunofluorescence, ELISA, culture | Blood, placenta, kidney, adrenal glands, intestines | *L. interrogans* serovar Hardjo | Initial symptomatic treatment and recovery of mother who recovered and later relapsed. Induced labour after intrauterine death. | Strong |
| Gsell et al., 1971 [5] | Switzerland | Vertical (transplacental) | MAT, 2-mercaptoethanol IgM assay | Serum | *L. interrogans* serovar Pomona | MAT, ELISA, Immunofluorescence test | Serum, urine | *L. interrogans* serovar Pomona | Neonate treated using ampicillin and recovered. No maternal interventions or outcome specified. | Strong |
| Hope et al., 2022 [6] | Uganda | Vertical (transplacental) | Not performed | NA | NA | IgM ELISA, LipL32 qPCR | Plasma | Pathogenic *Leptospira* spp. | 13.7% of neonates were on unspecified antibiotics. | Strong |
| Lindsay and Luke, 1949 [7] | United States of America | Vertical (transplacental) | Agglutination test | Blood | *L. interrogans* serovar Icterohaemorrhagiae, *L. interrogans* serovar Canicola | Histopathology, silver staining (Dieterle/Levaditi method) | Liver, kidney | *Leptospira* spp. | Neonate breastfed shortly before death. Mother had complete recovery without specific interventions. Mother had a subsequent uneventful pregnancy with negative serology results from her and the neonate. | Strong |
| Schiff et al., 2016 [8] | Uganda | Vertical (transplacental) | Not performed | NA | NA | 16S PCR, culture and Gram staining | Blood, cerebrospinal fluid | *L. broomii* or *L. inadai* | Unspecified | Strong |
| Teng et al., 1960 [9] | China | Vertical (transplacental) | Microscopy | Blood | *Leptospira* spp. | Microscopy, animal inoculation | Placenta | *Leptospira* spp. | Both mother and infant treated with penicillin and recovered. They were discharged after 12 days of hospitalisation. | Strong |
| Topciu et al., 1966 [10] | Romania | Vertical (transplacental) | Agglutination test, Levaditi silver staining, animal inoculation | Blood, liver | *Leptospira* spp. | Levaditi staining, animal inoculation | Liver | *Leptospira* spp. | No treatment specified for both mother and child. Mother died 7 hrs post-hospitalisation and foetus died too. | Strong |
| Coghlan and Bain, 1969 [11] | Scotland | Vertical (transplacental) | MAT, culture, animal inoculation | Serum, urine | *L. interrogans* serovar Canicola | Dark field microscopy, Silver-stained tissue sections (Warthin-Starry method), culture, and animal inoculation | Placental, heart, liver, kidney and lung tissues | No *Leptospira* isolated | No specific interventions mentioned for mother, but foetal death occurred during recovery. | Moderate |
| Colette, 1962 [12] | France | Vertical (transplacental) | Serology | Serum | *L. interrogans* serogroup Icterohaemorrhagiae | Pathological examination (macroscopy) | Placenta, foetal tissues | Presumed same group as maternal infection (serogroup Icterohaemorrhagiae) | Mother treated using penicillin and corticosteroids. No interventions for foetus mentioned. Both mother and child died. | Moderate |
| Gainder et al., 2010 [13] | India | Vertical (transplacental) | Culture, IgM ELISA | Blood, urine, serum | *L. interrogans* (serovar unspecified) | Not performed | NA | NA | Ceftriaxone and doxycycline, blood products transfusion and intensive supportive care to the mother who recovered. Intrauterine foetal death occurred. | Moderate |
| Aker et al., 1996 [14] | England | Vertical (transplacental) | MAT, IgM ELISA | Serum | *L. interrogans* serovar Hardjo | Immunofluorescence test | Unspecified foetal tissue | No leptospires confirmed | Mother treated for presumed UTI using amoxicillin before leptospirosis diagnosis. Induced labour after intrauterine death. | Weak |
| Bleier and Lechtken, 1951 [15] | Germany | Vertical (transplacental) | MAT | Urine, blood | *L. interrogans* serovar Canicola | MAT | Amniotic fluid | Unspecified | Spontaneous abortion at 18 weeks gestation. Mother recovered and was discharged. | Weak |
| Cárdenas-Marrufo et al., 2016 [16] | Mexico | Vertical (transplacental) | MAT, IgM ELISA, PCR | Serum | *L. kirschneri* serovar Cynopteri (9.1%), *L. interrogans* serovar Bratislava (9.1%), *L. santarosai* serovar Borincana (18.2%), *L. kirschneri* serovar Grippotyphosa (18.2%), *L. borgpetersenii* serovar Hardjo (45.5%) | 16S PCR | Paraffin-embedded placental tissue | No leptospires detected. Extracted DNA was degraded | The study was based on patients (81 women) who suffered from spontaneous abortions. No maternal or foetal interventions specified. | Weak |
| Carles et al., 1995  (Case 4)  [1] | French Guiana | Vertical (transplacental) | Culture, serology | Blood, urine | *L. interrogans* serovar Castellonis | Culture, serology | Placenta | Undetermined | Mother treated using ampicillin and gentamycin and pregnancy resulted in abortion. | Weak |
| Carles et al., 1995  (Case 8) [1] | French Guiana | Vertical (transplacental) | Culture, serology | Blood, urine | *L. interrogans* serovar Icterohaemorrhagiae | Culture, serology | Placenta | Undetermined | Maternal interventions and outcomes were unspecified. Intrauterine foetal death accompanied by foeto-placental anasarca occurred. | Weak |
| Carles et al., 1995 (Case 1) [1] | French Guiana | Vertical (transplacental) | Culture, serology | Blood, urine | *L. interrogans* serovar Australis | Culture, serology | Placenta | Undetermined | Mother treated using penicillin, and intrauterine foetal death accompanied by foeto-placental anasarca occurred. | Weak |
| Carles et al., 1995 (Case 2) [1] | French Guiana | Vertical (transplacental) | Culture, serology | Blood, urine | *L. interrogans* serovar Icterohaemorrhagiae | Culture, serology | Placenta | Undetermined | Mother treated using ampicillin, but pregnancy resulted in an intrauterine foetal death. | Weak |
| Carles et al., 1995 (Case 6) [1] | French Guiana | Vertical (transplacental) | Culture, serology | Blood, urine | *L. interrogans* serovar Grippotyphosa | Culture, serology | Placenta | Undetermined | Mother treated using ampicillin, but pregnancy resulted in an abortion. | Weak |
| Rahimi et al., 2018 [17] | Malaysia | Vertical (transplacental) | IgM serology, PCR | Blood, liver tissue | *Leptospira* spp. | NA | NA | NA | Emergency care to mother before she succumbed from multiple organ failure with septicaemia secondary to leptospirosis. Foetus died too. | Weak |
| Salgado et al., 1996 [18] | Cuba | Vertical (transplacental) | Unclear whether testing was performed | NA | *L. interrogans* serovar Canicola found from dog in patient’s home | Not performed | NA | NA | Critical care support, incl. antibiotic therapy (ceftriaxone, vancomycin, amikacin) to mother who recovered after 24 days but with spontaneous abortion of macerated foetus. | Weak |
| Sharma et al., 2011 [19] | India | Vertical (transplacental) | MAT, culture | Blood | *L. interrogans* serovar Copenhageni | Not performed | NA | NA | Cefoperazone/sulbactam, metronidazole, blood transfusion, and critical care support on the mother who recovered after 6 months of follow-up. Pregnancy resulted in still birth. | Weak |
| Shubhra et al., 2022 [20] | India | Vertical (transplacental) | IgM serology, culture | Serum, blood, ascitic fluid | *Leptospira* spp. | Not performed | NA | NA | Meropenem, Clindamycin and plasma transfusion for mother. Tigecycline used for nosocomial Klebsiella infection. No foetal interventions specified due to demise. | Weak |
| Suzuki, 1997 [21] | Laos | Vertical (transplacental) | Culture, MAT | Blood | *L. interrogans* serovar Autumnalis | Not performed | NA | NA | Antibiotic therapy (ampicillin, gentamicin and tetracycline) and supportive maternal care who was discharged in serious state and died at home. Foetus died during mother’s acute illness and still born was induced. | Weak |
| Udawat et al., 2014 [22] | India | Vertical (transplacental) | MAT, Macroscopic Slide Agglutination Test (MSAT) | Blood | *L. interrogans* serovar Australis (18 cases), *L. interrogans* serovar Icterohaemorrhagiae (11 cases), *L. interrogans* serovar Grippotyphosa (4 cases) | Not performed | NA | NA | Stillbirths (6), premature rupture of membranes (2), preterm birth (1), uneventful (24) in pregnant women with probable leptospirosis. | Weak |
| Bolin and Koellner, 1988 [23] | United States of America | Vertical (lactation) | MAT, culture | Urine, serum | *L. interrogans* serovar Hardjo | Urine culture, fluorescent antibody test | Urine | *L. interrogans* serovar Hardjo | Antibiotic therapy (penicillin G, then oral amoxicillin) to infant who recovered. Infant acquired infection from infected mother who breastfed throughout her illness. | Strong |
| Chung et al., 1963  (Case 3) [2] | China | Vertical (lactation) | culture, animal inoculation | Blood, milk | *L. interrogans* serovar Australis | Not performed | NA | NA | Unspecified interventions and outcome for both mother and child. | Weak |
| Doeleman, 1932 [24] | Netherlands | Sexual | Serology | Serum | *Leptospira* spp. | Unspecified | Blood | Unspecified | Unspecified interventions for both husband (index case) and wife (secondary case). The wife made a recovery. | Moderate |
| Szalka and Binder, 1974 [25] | Hungary | Sexual | Serology | Blood | *Leptospira serovars* Sejroe, Saxkoebing, and Mini | Unspecified | Blood | *Leptospira serovars* Sejroe, Saxkoebing, and Mini | Supportive care to husband (secondary case) after which he recovered after 19 days of hospitalisation. The wife (index case) was on antibiotics (tetracycline) and supportive care, and she recovered after 32 days. | Moderate |
| Harrison and Fitzgerald, 1988 [26] | West Germany | Sexual | MAT, IgM ELISA | Blood | *L. interrogans* serovar Icterohaemorrhagiae | MAT, ELISA IgM | Blood | *L. interrogans* serogroup Icterohaemorrhagiae | Husband (index case) and wife (secondary case) recovered spontaneously without any specific treatment. | Weak |
| Pons et al., 2016 [27] | Peru | Blood donation | PCR | Blood | pathogenic *Leptospira* | Not performed | NA | NA | Unspecified. | Weak |

* MAT testing is not definitive proof of infective serovars due to cross-reactivity between serovars.

CFT: complement fixation test; MAT: microscopic agglutination test; ELISA: enzyme-linked immunosorbent assay; PCR: polymerase chain reaction

# **JBI Quality assessment**

# **Supplementary references**

1. Carles G, Montoya E, Joly F, Peneau C. [Leptospirosis and pregnancy. Eleven cases in French Guyana]. J Gynecol Obstet Biol Reprod (Paris). 1995;24(4):418-21. PubMed Central PMCID: PMC7650320.

2. Chung H-L, TS'AO W-C, Mo Pe-s, YEN C. Transplacental or congenital infection of leptospirosis: clinical and experimental observations. Chinese Medical Journal. 1963;82(12):777-82. PubMed Central PMCID: PMC14100807.

3. Cramer H, Wadulla H. Abortus bei Leptospirosis canicola. Archiv für Gynäkologie. 1950;177(2):167-77. doi: 10.1007/BF00981250.

4. Faine S, Valentine R. Leptospirosis hardjo in pregnancy. Medical Journal of Australia. 1984;140(5):311-2. doi: https://doi.org/10.5694/j.1326-5377.1984.tb104077.x.

5. Gsell HO, Jr., Olafsson A, Sonnabend W, Breer C, Bachmann C. [Intrauterine leptospirosis pomona. 1st reported case of an intrauterine transmitted and cured leptospirosis]. Deutsche Medizinische Wochenschrift. 1971;96(31):1263-8. doi: https://doi.org/10.1055/s-0028-1110120. PubMed Central PMCID: PMC4934402.

6. Hope D, Businge S, Kyoyagala S, Bazira J. Prevalence of anti-leptospiral IgM and detection of pathogenic *Leptospira* species DNA in neonates presenting with clinical sepsis in Southwestern Uganda. European Journal of Medical Research. 2022;27(1):268. doi: https://doi.org/10.1186/s40001-022-00902-w.

7. Lindsay S, Luke IW. Fatal leptospirosis (Weil's disease) in a newborn infant: Case of intrauterine fetal infection with report of an autopsy. The Journal of Pediatrics. 1949;34(1):90-4. doi: https://doi.org/10.1016/S0022-3476(49)80206-X.

8. Schiff SJ, Kiwanuka J, Riggio G, Nguyen L, Mu K, Sproul E, et al. Separating putative pathogens from background contamination with principal orthogonal decomposition: Evidence for *Leptospira* in the Ugandan neonatal septisome. Front Med (Lausanne). 2016;3:22. Epub 20160613. doi: https://doi.org/10.3389/fmed.2016.00022. PubMed PMID: 27379237; PubMed Central PMCID: PMCPMC4904006.

9. Teng C. Fetus with leptospirosis infection during pregnancy: a case report. Zhong Neike Z. 1960;8(291).

10. Topciu V, Manu E, Strubert L, Duma G, Levin S. Voie transplacentaire dans un cas de leptospirose humaine. Gynecol Obstet (Paris). 1966;65:617-20.

11. Coghlan JD, Bain AD. Leptospirosis in human pregnancy followed by death of the foetus. British Medical Journal. 1969;1(5638):228-30. doi: https://doi.org/10.1136/bmj.1.5638.228.

12. Colette C. [Fatal leptospirosis during pregnancy with severe jaundice]. Bull Fed Soc Gynecol Obstet Lang Fr. 1962;14:437-40. PubMed Central PMCID: PMC14022281.

13. Gainder S, Singla R, Dhaliwal L, Suri V. Leptospirosis as a cause of intrauterine fetal demise: short report of rare presentation. Archives of Gynecology and Obstetrics. 2010;281(6):1061-3. doi: https://doi.org/10.1007/s00404-009-1266-y.

14. Aker N, James EB, Johnston AM, Pasvol G. Leptospirosis in pregnancy: An unusual and relatively unrecognised cause of intrauterine death in man. Journal of Obstetrics and Gynaecology. 1996;16(3):163-5. doi: https://doi.org/10.3109/01443619609004093.

15. Bleier W, Lechtken F. [Leptospirosis canicola in man as a cause of spontaneous abortion]. Geburtshilfe und Frauenheilkunde. 1951;11(6):538-44. PubMed Central PMCID: PMC 14849774.

16. Cárdenas-Marrufo MF, Vado-Solis I, Peniche-Lara GF, Perez-Osorio C, Correa-Segura J. A cross sectional study of leptospirosis and fetal death in Yucatan, Mexico. Colombia Medica. 2016;47(1):11-4. doi: https://doi.org/10.25100/cm.v47i1.1975.

17. Rahimi R, Omar E, Soh TST, Nawi SFAM, Noor SM. Leptospirosis in pregnancy: A lesson in subtlety. The Malaysian Journal of Pathology. 2018;40(2):169-73. PubMed Central PMCID: PMC30173235.

18. Trujillo Salgado JE, Martínez Torres A, Mármol Sóñora A. Leptospirosis, enfermedad de Weil y falla multiorgánica: Informe de 1 caso. Revista Cubana de Medicina. 1996;35(3):212-5.

19. Sharma KK, Madhvilatha P, Kalawat U, Sivakumar V. Leptospirosis-induced still birth and postpartum sepsis. Indian Journal of Pathology and Microbiology. 2011;54(2). doi: https://doi.org/10.4103/0377-4929.81617.

20. Shubhra, Yadav A, Goila AK, Kaur R. Management of leptospirosis in postpartum period in ICU. Saudi J Anaesth. 2022;16(1):130-1. Epub 20220104. doi: https://doi.org/10.4103/sja.sja_556_21. PubMed PMID: 35261607; PubMed Central PMCID: PMCPMC8846237.

21. Suzuki K, Nakamura S, Watanabe H. A fatal case of *Leptospira autumnalis* infection in Lao PDR. Southeast Asian J Trop Med Public Health. 1997;28(2):436-7. PubMed Central PMCID: PMC9444037.

22. Udawat M, Sumathi G, Nithyalakshmi J, Krishnan M. Clinical impact of leptospirosis on pregnancy: an analysis. J Evol Med Dent Sci. 2014;3(58):13071-8. doi: https://doi.org/10.14260/jemds/2014/3738.

23. Bolin CA, Koellner P. Human-to-human transmission of *Leptospira interrogans* by milk. The Journal of Infectious Diseases. 1988;158(1):246-7. doi: https://doi.org/10.1093/infdis/158.1.246.

24. Doeleman F. Ziekte van Weil, rechstreeks overgebracht van mensch op mensch. Ned Tijdschr Geneeskd. 1932;76:5057-8.

25. Szalka A, Binder L. [A rare case of human-to-human infection of leptospirosis]. Orvosi Hetilap. 1974;115(26):1531-2. PubMed Central PMCID: PMC4840706.

26. Harrison NA, Fitzgerald WR. Leptospirosis — can it be a sexually transmitted disease? Postgraduate Medical Journal. 1988;64(748):163-4. doi: https://doi.org/10.1136/pgmj.64.748.163.

27. Pons MJ, Urteaga N, Alva-Urcia C, Lovato P, Silva J, Ruiz J, et al. Infectious agents, *Leptospira* spp. and *Bartonella* spp., in blood donors from Cajamarca, Peru. Blood Transfusion. 2016;14(6):504-8. Epub 20151201. doi: https://doi.org/10.2450/2015.0081-15. PubMed PMID: 26674831; PubMed Central PMCID: PMCPMC5111371.

# **PRISMA Checklist**

| 1. **Section and Topic** | **Item #** | **Checklist item** | **Location where item is reported** |
| --- | --- | --- | --- |
| **TITLE** | | |  |
| Title | 1 | Identify the report as a systematic review. | Title |
| **ABSTRACT** | | |  |
| Abstract | 2 | See the PRISMA 2020 for Abstracts checklist. | Abstract |
| **INTRODUCTION** | | |  |
| Rationale | 3 | Describe the rationale for the review in the context of existing knowledge. | Introduction |
| Objectives | 4 | Provide an explicit statement of the objective(s) or question(s) the review addresses. | Introduction |
| **METHODS** | | |  |
| Eligibility criteria | 5 | Specify the inclusion and exclusion criteria for the review and how studies were grouped for the syntheses. | Methods |
| Information sources | 6 | Specify all databases, registers, websites, organisations, reference lists and other sources searched or consulted to identify studies. Specify the date when each source was last searched or consulted. | Methods |
| Search strategy | 7 | Present the full search strategies for all databases, registers and websites, including any filters and limits used. | Supp. Mat. |
| Selection process | 8 | Specify the methods used to decide whether a study met the inclusion criteria of the review, including how many reviewers screened each record and each report retrieved, whether they worked independently, and if applicable, details of automation tools used in the process. | Methods |
| Data collection process | 9 | Specify the methods used to collect data from reports, including how many reviewers collected data from each report, whether they worked independently, any processes for obtaining or confirming data from study investigators, and if applicable, details of automation tools used in the process. | Methods |
| Data items | 10a | List and define all outcomes for which data were sought. Specify whether all results that were compatible with each outcome domain in each study were sought (e.g. for all measures, time points, analyses), and if not, the methods used to decide which results to collect. | Methods |
|  | 10b | List and define all other variables for which data were sought (e.g. participant and intervention characteristics, funding sources). Describe any assumptions made about any missing or unclear information. | Methods, Supp. Mat. |
| Study risk of bias assessment | 11 | Specify the methods used to assess risk of bias in the included studies, including details of the tool(s) used, how many reviewers assessed each study and whether they worked independently, and if applicable, details of automation tools used in the process. | Methods |
| Effect measures | 12 | Specify for each outcome the effect measure(s) (e.g. risk ratio, mean difference) used in the synthesis or presentation of results. | NA |
| Synthesis methods | 13a | Describe the processes used to decide which studies were eligible for each synthesis (e.g. tabulating the study intervention characteristics and comparing against the planned groups for each synthesis (item #5)). | NA |
|  | 13b | Describe any methods required to prepare the data for presentation or synthesis, such as handling of missing summary statistics, or data conversions. | NA |
|  | 13c | Describe any methods used to tabulate or visually display results of individual studies and syntheses. | NA |
|  | 13d | Describe any methods used to synthesize results and provide a rationale for the choice(s). If meta-analysis was performed, describe the model(s), method(s) to identify the presence and extent of statistical heterogeneity, and software package(s) used. | NA |
|  | 13e | Describe any methods used to explore possible causes of heterogeneity among study results (e.g. subgroup analysis, meta-regression). | NA |
|  | 13f | Describe any sensitivity analyses conducted to assess robustness of the synthesized results. | NA |
| Reporting bias assessment | 14 | Describe any methods used to assess risk of bias due to missing results in a synthesis (arising from reporting biases). | NA |
| Certainty assessment | 15 | Describe any methods used to assess certainty (or confidence) in the body of evidence for an outcome. | NA |
| **RESULTS** | | |  |
| Study selection | 16a | Describe the results of the search and selection process, from the number of records identified in the search to the number of studies included in the review, ideally using a flow diagram. | Results |
|  | 16b | Cite studies that might appear to meet the inclusion criteria, but which were excluded, and explain why they were excluded. | Results |
| Study characteristics | 17 | Cite each included study and present its characteristics. | Results |
| Risk of bias in studies | 18 | Present assessments of risk of bias for each included study. | Results, Supp. Mat. |
| Results of individual studies | 19 | For all outcomes, present, for each study: (a) summary statistics for each group (where appropriate) and (b) an effect estimate and its precision (e.g. confidence/credible interval), ideally using structured tables or plots. | NA |
| Results of syntheses | 20a | For each synthesis, briefly summarise the characteristics and risk of bias among contributing studies. | NA |
|  | 20b | Present results of all statistical syntheses conducted. If meta-analysis was done, present for each the summary estimate and its precision (e.g. confidence/credible interval) and measures of statistical heterogeneity. If comparing groups, describe the direction of the effect. | NA |
|  | 20c | Present results of all investigations of possible causes of heterogeneity among study results. | NA |
|  | 20d | Present results of all sensitivity analyses conducted to assess the robustness of the synthesized results. | NA |
| Reporting biases | 21 | Present assessments of risk of bias due to missing results (arising from reporting biases) for each synthesis assessed. | NA |
| Certainty of evidence | 22 | Present assessments of certainty (or confidence) in the body of evidence for each outcome assessed. | NA |
| **DISCUSSION** | | |  |
| Discussion | 23a | Provide a general interpretation of the results in the context of other evidence. | Discussion |
|  | 23b | Discuss any limitations of the evidence included in the review. | Discussion |
|  | 23c | Discuss any limitations of the review processes used. | Discussion |
|  | 23d | Discuss implications of the results for practice, policy, and future research. | Discussion |
| **OTHER INFORMATION** | | |  |
| Registration and protocol | 24a | Provide registration information for the review, including register name and registration number, or state that the review was not registered. | Methods |
|  | 24b | Indicate where the review protocol can be accessed, or state that a protocol was not prepared. | Methods |
|  | 24c | Describe and explain any amendments to information provided at registration or in the protocol. | Methods |
| Support | 25 | Describe sources of financial or non-financial support for the review, and the role of the funders or sponsors in the review. | Funding |
| Competing interests | 26 | Declare any competing interests of review authors. | Competing interests |
| Availability of data, code and other materials | 27 | Report which of the following are publicly available and where they can be found: template data collection forms; data extracted from included studies; data used for all analyses; analytic code; any other materials used in the review. | Data availability |

*From:*  Page MJ, McKenzie JE, Bossuyt PM, Boutron I, Hoffmann TC, Mulrow CD, et al. The PRISMA 2020 statement: an updated guideline for reporting systematic reviews. BMJ 2021;372:n71. doi: 10.1136/bmj.n71. This work is licensed under CC BY 4.0. To view a copy of this license, visit <https://creativecommons.org/licenses/by/4.0/>
